# Supplementary material for: Sequential buckling in fluid-filled cylindrical shells
Source: Commun Phys. 2026 Mar 31;9(1):114. doi: 10.1038/s42005-026-02589-5 (PMC13098793; doi:10.1038/s42005-026-02589-5)
Supplement: Supplementary file 2 — Supplementary Information [file 42005_2026_2589_MOESM2_ESM.pdf]

Supplementary information for the manuscript "Sequential buckling in fluid-filled cylindrical shells"

Shresht Jain[1,2], Finn Box[1,2], Martin Quinn[1,2], Chris Johnson[2,3], and Draga Pihler-Puzović[1,2]

[1] *Physics of Fluids & Soft Matter, Department of Physics & Astronomy, University of Manchester, Manchester M13 9PL, UK*

[2] *Manchester Centre for Nonlinear Dynamics, University of Manchester, Manchester M13 9PL, UK and*

[3] *Department of Mathematics, University of Manchester, Manchester M13 9PL, UK*

Supplementary Note 1: Profile Extraction Procedure

During the compression tests, we imaged the profiles of the samples using a Nikon D7500 DSLR camera. The tests were recorded at 25 fps. The cans were imaged with a contrasting background for edge detection in MATLAB. We measured the length and diameter of the can before compression to calibrate pixel resolution. The colour images were converted to grey scale and then binarised by thresholding. Finally, can profiles were extracted by tracking the pixel positions where the pixel intensity values change from zero to one. This procedure was used to find can profiles such as the one shown in Fig. 1a of the main manuscript.

Supplementary Note 2: Experimental data from Fig. 2b of the main manuscript.

Test Geometry ($\sqrt{Rt}$ (mm))	Shell Thickness (t (mm))	Pattern Wavelength ($\langle\lambda\rangle$ (mm))	Number of Repeated Tests	Saturation Amplitude ($\langle\, w_{\max}\, \rangle$ (mm))
1.543 (Pressurised)	0.1	11.86 ± 0.95	2	Samples exploded
1.543 (Unpressurised)	0.1	11.80 ± 1.80	6	2.53 ± 0.42
1.81 (Pressurised)	0.1	10.54 ± 1.94	2	1.41 ± 0.21
1.99 (Unpressurised)	0.15	15.6 ± 1.88	2	4.11 ± 1.1
2.54 (Pressurised)	0.15	19.53 ± 3.05	2	6.79 ± 0.86
2.91 (Unpressurised)	0.2	22.92 ± 3.97	1	7.42 ± 0.75
3.1 (Unpressurised)	0.22	18.70 ± 0.81	1	6.49 ± 1.02

TABLE S1: Experimental data from Fig. 2b of the main manuscript, which also includes the number of repeated tests for each data point. For each geometry, the errors on the wavelength cited in the second column, and shown as error bars in Fig. 2b of the main manuscript, correspond to the standard deviation of the peak-to-peak separation between can undulations across the peaks of all samples. The saturation amplitude was determined by measuring a mean distance between the peak and the trough of undulations and the standard deviation of this quantity in a representative sample. We have independently verified that samples with the same geometry had consistent measurements.

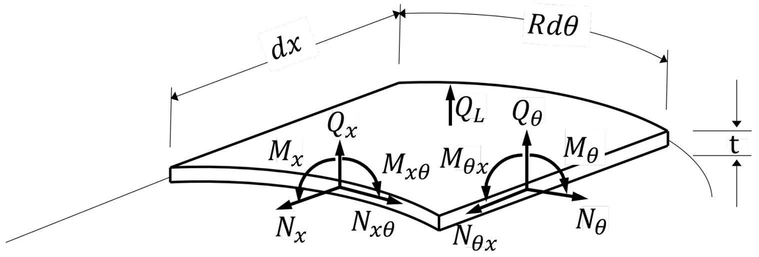

FIG. S1: **Shell diagram.** An element of the cylindrical shell, on which the notation for the forces and moments is defined.

Supplementary Note 3: Deriving Axi-symmetric Shell Equations

Our cans are thin-walled cylinders under axial compression. We model them as cylindrical shells of length L, wall thickness t and initial radius R, such that $t \ll R \sim L$, loaded with an internal pressure Q_L, and an axial load F. Following [1] and [2], and assuming axi-symmetric deformations, we derive the kinematic relations and the force and moment balance equations for the middle surface of this shell.

We define a cylindrical coordinate system (x, θ, r) centred around the axis of rotation of the shell. The internal stresses within the shell $\{\bar{\sigma}_x, \bar{\sigma}_\theta, \bar{\tau}_{x\theta}, \bar{\tau}_{\theta x}, \bar{\tau}_{xr}, \bar{\tau}_{\theta r}\}$ are linked to the force and moment intensities $\{N_x, N_\theta, N_{x\theta}, N_{\theta x}, Q_x, Q_\theta, M_x, M_\theta, M_{x\theta}, M_{\theta x}\}$ illustrated in Fig. S1 as follows:

$$\begin{aligned} N_x &= \int_{R-t/2}^{R+t/2} \bar{\sigma}_x \left(1+\frac{r}{R}\right) dr,\ N_\theta = \int_{R-t/2}^{R+t/2} \bar{\sigma}_\theta dr, \\ N_{x\theta} &= \int_{R-t/2}^{R+t/2} \bar{\tau}_{x\theta} \left(1+\frac{r}{R}\right) dr,\ N_{\theta x} = \int_{R-t/2}^{R+t/2} \bar{\tau}_{\theta x} dr, \\ Q_x &= \int_{R-t/2}^{R+t/2} \bar{\tau}_{xr} \left(1+\frac{r}{R}\right) dr,\ Q_\theta = \int_{R-t/2}^{R+t/2} \bar{\tau}_{\theta r} dr, \end{aligned} \tag{S1}$$

$$\begin{aligned} M_x &= R\int_{R-t/2}^{R+t/2} \bar{\sigma}_x \left(1+\frac{r}{R}\right) r dr,\ M_\theta = R\int_{R-t/2}^{R+t/2} \bar{\sigma}_\theta r dr, \\ M_{x\theta} &= R\int_{R-t/2}^{R+t/2} \bar{\tau}_{x\theta} \left(1+\frac{r}{R}\right) r dr,\ M_{\theta x} = R\int_{R-t/2}^{R+t/2} \bar{\tau}_{\theta x} r dr. \end{aligned} \tag{S2}$$

In the limit of small displacements (relative to the shell radius), the force balance equations are:

$$\begin{aligned}\frac{dN_x}{dx} + \frac{1}{R}\frac{dN_{\theta x}}{d\theta} &= 0,\\ \frac{dN_\theta}{dx} + \frac{1}{R}\frac{dN_\theta}{d\theta} &= 0.\end{aligned} \tag{S3}$$

We also obtain the (general) moment balance equation:

$$\frac{d^2M_x}{dx^2} + \frac{2}{R}\frac{d^2M_{x\theta}}{dxd\theta} + \frac{1}{R^2}\frac{d^2M_\theta}{d\theta^2} + \frac{N_\theta}{R} - N_x\frac{d^2w}{dx^2} - 2\frac{N_{x\theta}}{R}\frac{d^2w}{d\theta dx} - \frac{N_\theta}{R^2}\frac{d^2w}{d\theta^2} = Q_L. \tag{S4}$$

Assuming (u, v, w) are displacements in (x, θ, r), respectively, we write the kinematic relations on the middle surface of the shell for the extensional and flexural strains as:

$$\begin{aligned}\varepsilon_x &= \frac{du}{dx} + \frac{1}{2}\left(\frac{dw}{dx}\right)^2, \quad \kappa_x = \frac{d^2w}{dx^2},\\ \varepsilon_\theta &= \frac{1}{R}\frac{dv}{d\theta} + \frac{w}{R} + \frac{1}{2R^2}\left(\frac{dw}{d\theta}\right)^2, \quad \kappa_\theta = \frac{1}{R^2}\frac{d^2w}{d\theta^2},\\ \varepsilon_{x\theta} &= \frac{1}{R}\frac{du}{d\theta} + \frac{dv}{dx} + \frac{1}{R}\frac{dw}{d\theta}\frac{dw}{dx}, \quad \kappa_{x\theta} = \frac{1}{R}\frac{d^2w}{dxd\theta}.\end{aligned} \tag{S5}$$

If all displacements are axi-symmetric, i.e. $\frac{du}{d\theta} = \frac{dv}{d\theta} = \frac{dw}{d\theta} = v = 0$, and assuming small curvatures, the kinematic relations Eq. (S5) simplify to:

$$\begin{aligned}&\varepsilon_x = \frac{du}{dx} + \frac{1}{2}\left(\frac{dw}{dx}\right)^2, \quad \varepsilon_\theta = \frac{w}{R}, \quad \varepsilon_{x\theta} = 0,\\ &\kappa_x = \frac{d^2w}{dx^2}, \quad \kappa_\theta = 0, \quad \kappa_{x\theta} = 0,\end{aligned} \tag{S6}$$

so only the extensional strains in the axial and circumferential directions, and the axial curvature are non-zero, respectively. Similarly, due to axi-symmetry, the force and moment balance equations (S3) and (S4) reduce to:

$$\begin{aligned}&\frac{dN_x}{dx} = 0,\\ &\frac{d^2M_x}{dx^2} + \frac{N_\theta}{R} - N_x\frac{d^2w}{dx^2} = Q_L.\end{aligned} \tag{S7}$$

Our cans exhibit nonlinear material properties, so we will model the constitutive relations governing their middle surface as

$$\begin{aligned}M_x &= M_x(\kappa_x),\\ N_x &= C_x\varepsilon_x + \nu C_\theta(\varepsilon_\theta)\varepsilon_\theta,\\ N_\theta &= \nu C_x\varepsilon_x + C_\theta(\varepsilon_\theta)\varepsilon_\theta,\end{aligned} \tag{S8}$$

where $M_x(\kappa_x)$ and $C_\theta(\varepsilon_\theta)$ are nonlinear functions of respective variables that we will determine experimentally, C_x is the extensional stiffness intensity in the x direction and ν is the Poisson's ratio. Note that Eq. (S8) also assumes an anisotropic response in the axial and circumferential directions; this anisotropy is further stressed by the constraints discussed in Supplementary Note 5. We note that the stretching stiffness in the x-direction (C_x) will not feature in the final system of equations, but $M_x(\kappa_x)$ and $C_\theta(\varepsilon_\theta)$ need to be determined as discussed in the following section.

Supplementary Note 4: Measuring Constitutive Response

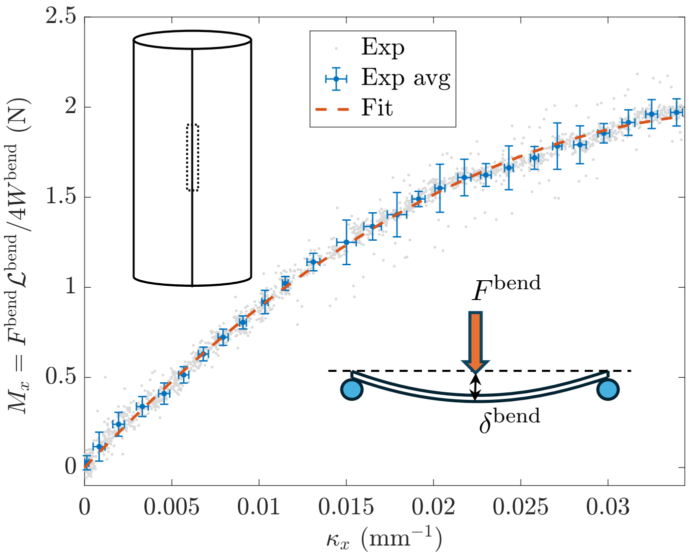

FIG. S2: **Three-point bend tests.** Bending moment intensity $M_x = F^{\text{bend}}\mathcal{L}^{\text{bend}}/4W^{\text{bend}}$ as a function of the curvature of the beam κ_x at the point of the maximum deflection δ^{bend}, the average of these measurements and the quadratic fit to the average given by Eq. (S9), as indicated in the legend. Test samples were aluminium strips, orientated in the axial direction (top inset), cut out from commercial cans. They were simply supported at their two end points and subjected to the control displacement δ^{bend} at the mid-point as shown in the bottom inset, while the corresponding force F^{bend} and resulting shape were measured.

Most of our experiments were performed using aluminium cans. Aluminium is well known for its yielding behaviour and plastic deformations under moderate strains. The cans in our compression tests are deformed beyond the yielding threshold and have a nonlinear

constitutive response of the material.

We determined the nonlinear material response of the cans in axial bending and circumferential stretching by testing rectangular samples cut from can walls in the respective directions. We subjected these samples to (quasi-static) three-point bend and extensional tests using a Universal Testing System (Instron 3345) with a 1 kN load cell. The results from these tests are shown in Figs. S2 and S3, where the insets show schematic diagrams of the tested samples and the corresponding tests.

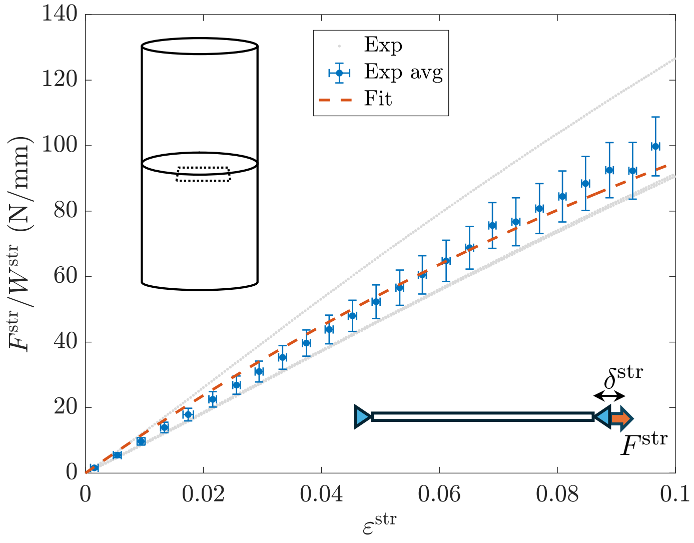

FIG. S3: **Extensional tests.** Experimental measurements of the scaled force $F^{\mathrm{str}}/W^{\mathrm{str}}$ as a function of increasing engineering strain $\varepsilon^{\mathrm{str}} = \delta^{\mathrm{str}}/\mathcal{L}^{\mathrm{str}}$ in extensional tests, the average of these measurements and the cubic fit to the average given by Eq. (S10), as indicated in the legend. Test samples were aluminium strips cut out from a circumference of the can (top inset) of length $\mathcal{L}^{\mathrm{str}}$ that were stretched by δ^{str}, as shown in the bottom inset.

When performing the three-point bend test, we placed a sample on two supports and applied a controlled displacement δ^{bend} to its mid-point, recording the resulting force F^{bend} and the shape of the sample as it deformed. The latter was done by illuminating the sample edge with a laser line and imaging the deformation profile of the edge from below using a HD camera (Nikon D7500, 1920 pixels $\times$ 1080 pixels resulting in the resolution of 57.7 μm per pixel) oriented at an angle of $\theta = 8.3°$ to the horizontal. The sample deflection was then determined by reference to the initial, undeformed position of the edge before the sample was deformed and rescaled by a trigonometric factor of $1/\cos(\theta)$, see [3]. Once the profiles were

reconstructed, we fitted (symmetric) fourth-order polynomials to these data and computed the axial curvature κ_x of the deforming samples at the point of maximum deflection. We tested samples for κ_x up to 0.035 mm^{-1}, which was the range that we could reliably test with our setup.

The raw data from three repeated measurements with samples of length $\mathcal{L}^{\text{bend}} = 29.8$ mm and width in the range $2 \leq W^{\text{bend}} \leq 5$ mm are shown with (gray) dotted lines in Fig. S2, where the measured force F^{bend} is related to the bending moment per unit width M_x as $F^{\text{bend}}\mathcal{L}^{\text{bend}}/4W^{\text{bend}}$. We find the average of these data by binning all curvatures into n bins of varying widths, where n equals to the smallest number of recorded data points for a single test and each bin contains the same amount of data. The reported average curvature is then the central value of the bin, and the errors are given by half of the bin width. For each value of the average curvature, the average value of M_x was obtained by finding the mean of all data points contained within the bin, with the error taken to be a quarter of the range of values recorded within the bin. The resulting data are plotted using (blue) markers with error bars in the same figure. The measurements suggest that the moment intensity is a nonlinear function of the curvature. We assume a quadratic dependence and fit

$$M_x = D(\kappa_x + \alpha\kappa_x^2), \tag{S9}$$

to the data. The result of this fit is shown using a (red) dashed line in Fig. S2. The values for D and α determined from fits to experimental data are reported in Table S2. We ascribe the nonlinearities in (S9) to material yielding because the samples were observed to permanently deform during the tests. While the material response to bending of the entire fluid-filled cans is likely different from that of the cutout strips, we believe that testing samples of the can wall still provides a solid estimate of this bending behaviour, and therefore a valid starting point for our modelling.

The extensional tests were performed by stretching samples, from the circumference of the can, of length $\mathcal{L}^{\text{str}} = 5$ mm and width $W^{\text{str}} = 3$ mm. We straightened the samples before they were loaded into a tensile test rig, which required a force of a few orders of magnitude smaller than in the extensional experiments. We applied a displacement δ^{str} and measured the resultant force F^{str} and show its dependence on the applied engineering strain, i.e. $\varepsilon^{\text{str}} = \delta^{\text{str}}/\mathcal{L}^{\text{str}}$, in Fig. S3, where (gray) dotted lines are used for raw data from four repeated tests and (blue) markers with error bars are the average data obtained following

the same binning procedure as described above. We fit the relationship

$$F^{\rm str} = H(\varepsilon + \gamma_1 \varepsilon^{\rm str 2} + \gamma_2 \varepsilon^{\rm str 3}), \tag{S10}$$

to this average, with (red) dashed line showing the result of fitting in Fig. S3. Since the extensional rigidity of the shell circumference is defined as the force per unit width for an applied strain, it follows that

$$C_\theta = \frac{F^{\rm str}}{(1-\nu^2) W^{\rm str} \varepsilon^{\rm str}},$$

where the azimuthal and axial force intensities are linked as

$$N_\theta = \nu N_x + (1-\nu^2) C_\theta(\varepsilon_\theta)\varepsilon_\theta, \tag{S11}$$

see Eq. (S8). Thus,

$$C_\theta = C(1 + \gamma_1 \varepsilon_\theta + \gamma_2 \varepsilon_\theta^2), \tag{S12}$$

where

$$C = \frac{H}{(1-\nu^2)\, W^{\rm str}}.$$

The values of C, γ_1 and γ_2 are obtained from the fit shown in Fig. S3 with (red) dashed line and are reported in Table S2.

Parameter	Measured Value
D	102.15 ± 0.59 N mm
α	-12.84 ± 0.19 mm
C	860 ± 32 N/mm
γ_1	-2.72 ± 0.17
γ_2	2.96 ± 0.43

TABLE S2: Measured values of coefficients that describe the nonlinear material response.

Supplementary Note 5: Closing the Theoretical Model

Returning to the can compression experiment, we assume that all of the axial force intensity N_x is supplied by the axial loading force. Then, from Eq. (S7) it follows that

$$N_x = -\frac{F}{2\pi R}, \tag{S13}$$

and by combining Eqs. (S5)-(S13), we obtain

$$
\begin{aligned}
&D\left[\frac{d^4w}{dx^4}\left(1+2\alpha\frac{d^2w}{dx^2}\right)+2\alpha\left(\frac{d^3w}{dx^3}\right)^2\right] \\
&+\frac{F}{2\pi R}\frac{d^2w}{dx^2}-\nu\frac{F}{2\pi R^2}+\frac{(1-\nu^2)C}{R}\left(\frac{w}{R}+\gamma_1\left(\frac{w}{R}\right)^2+\gamma_2\left(\frac{w}{R}\right)^3\right)=Q_L.
\end{aligned}
\tag{S14}
$$

However, this equation fails to link the level of can compression to its internal pressure Q_L and the measured force F; the theoretical model must be closed, therefore, by supplementing Eq. (S14) with additional integral constraints.

The first constraint relates changes in the can volume to its internal pressure. We assume that a fixed volume ratio of the can $\mathcal{I}$ is initially filled with an incompressible fluid, while the rest is filled with an ideal gas. Thus, the volume of the can that may be compressed at the start of the experiment is given by $(1-\mathcal{I})\,\pi R^2 L$. After compression starts, the can volume can be calculated as

$$\int_0^{L-\delta x}\pi(w+R)2dx,$$

where δx is the level of compression. Therefore, the compressible volume is given by

$$\int_0^{L-\delta x}\pi(w+R)^2dx-\mathcal{I}\pi R^2L,$$

and we can relate the pressure inside the can Q_L to its initial internal pressure Q_{i} using Boyle's law as

$$Q_L\left[\int_0^{L-\delta x}\pi(w+R)^2dx-\mathcal{I}\pi R^2L\right]=Q_{\mathrm{i}}\left[1-\mathcal{I}\right]\pi R^2L. \tag{S15}$$

Finally, once again we enforce an anisotropic response of the can, and assume that its axial length remains unchanged,

$$\int_0^{L-\delta x}\sqrt{1+\left(\frac{dw}{dx}\right)^2}dx=L. \tag{S16}$$

This assumption has strong implications, enforcing the anisotropy already underpinned by the nonlinear response of the can material to axial bending and circumferential stretching, which was determined empirically in the Supplementary Note 4. The axial coordinate is chosen to correspond to the directions in which the base metal is stretched during the die-forming production of cans. Hence, in addition to neglecting bending in the circumferential direction, we also assume that the cans do not stretch axially.

Therefore, our theoretical model is given by Eqs. (S14)–(S16) and is solved by applying simply supported boundary conditions,

$$\begin{aligned} &w(x=0) = w(x=L-\delta x) = 0, \\ &\frac{d^2w}{dx^2}(x=0) = \frac{d^2w}{dx^2}(x=L-\delta x) = 0. \end{aligned} \tag{S17}$$

The boundary conditions in our experiments are likely more complicated, since, for example, the commercially produced beverage cans do differ slightly in shape near the edges, with implications for the contact with the loading cell and their frictional response to it. This can in turn influence the pattern formation, for example, where the first buckle is localised on the surface. While there is an effect of boundaries on the pattern formation in the nonlinear SH equation [4], for the sake of simplicity, we proceed with simply supported boundary conditions given in (S17) and collect data that minimise the effect of boundaries (see, for example, Fig. 1b and the Results section of the main manuscript).

Supplementary Note 6: Numerical Implementation in AUTO-07p

The problem, i. e. Eqs. (S14)–(S17), is solved numerically by mapping the domain $x = [\![0, L-\delta x]\!]$ to a fixed domain $z = [\![-L/2, L/2]\!]$ and applying a collocation method. The mapping is given by the expression $z = ax + b$, where $a = (L-\delta x)/L$ and $b = -L/2$. Thus,

$$\frac{d}{dx} = \frac{d}{dz}\frac{dz}{dx} = a\frac{d}{dz}, \quad \text{and hence} \quad \frac{d^{\mathrm{m}}}{dx^{\mathrm{m}}} = a^{\mathrm{m}}\frac{d^{\mathrm{m}}}{dz^{\mathrm{m}}},$$

where m is the order of the derivative. When re-written in the new coordinate, our model becomes:

$$\begin{aligned} &D\left[a^4\frac{d^4w}{dz^4}\left(1+2\alpha a^2\frac{d^2w}{dz^2}\right)+2\alpha a^6\left(\frac{d^3w}{dz^3}\right)^2\right] \\ &+\frac{F}{2\pi R}a^2\frac{d^2w}{dz^2}-\nu\frac{F}{2\pi R^2}+\frac{(1-\nu^2)C}{R}\left(\frac{w}{R}+\gamma_1\left(\frac{w}{R}\right)^2+\gamma_2\left(\frac{w}{R}\right)^3\right)=Q_L, \\ &Q_L\left[\int_{-L/2}^{L/2}\pi(w+R)^2\frac{dz}{a}-\mathcal{I}\pi R^2 L\right]=Q_{\mathrm{i}}\left[1-\mathcal{I}\right]\pi R^2 L, \\ &\int_{-L/2}^{L/2}\sqrt{1+\left(a\frac{dw}{dz}\right)^2}\frac{dz}{a}=L, \\ &w(z=-L/2)=w(z=L/2)=0, \\ &\frac{d^2w}{dz^2}(z=-L/2)=\frac{d^2w}{dz^2}(z=L/2)=0. \end{aligned} \tag{S18}$$

Next we nondimensionalize Eq. (S18) using initial half-length of the can $L/2$ as a characteristic lengthscale, while scaling the deflections with the initial can radius R and choosing $DR/(L/2)^4$ as a pressure scale, to obtain

$$
\begin{aligned}
&a^4\left[\frac{d^4\mathcal{W}}{d\mathcal{Z}^4}\left(1+2a^2\beta\frac{d^2\mathcal{W}}{d\mathcal{Z}^2}\right)+2a^2\beta\left(\frac{d^3\mathcal{W}}{d\mathcal{Z}^3}\right)^2\right]+a^2\mathcal{F}\frac{d^2\mathcal{W}}{d\mathcal{Z}^2}-\frac{\nu\mathcal{F}}{\ell^2}+\mathcal{C}\left(\mathcal{W}+\gamma_1\mathcal{W}^2+\gamma_2\mathcal{W}^3\right)=\mathcal{P},\\
&\left[\int_{-1}^{1}(\mathcal{W}+1)^2\frac{d\mathcal{Z}}{2a}-\mathcal{I}\right]=\frac{\mathcal{P}_\mathrm{i}}{\mathcal{P}}\left[1-\mathcal{I}\right],\\
&\int_{-1}^{1}\sqrt{1+\left(a\ell\frac{d\mathcal{W}}{d\mathcal{Z}}\right)^2}\frac{d\mathcal{Z}}{a}=2,\\
&\mathcal{W}(\mathcal{Z}=-1)=\mathcal{W}(\mathcal{Z}=1)=0,\\
&\frac{d^2\mathcal{W}}{d\mathcal{Z}^2}(\mathcal{Z}=-1)=\frac{d^2\mathcal{W}}{d\mathcal{Z}^2}(\mathcal{Z}=1)=0,
\end{aligned}
\tag{S19}
$$

where

$$\mathcal{Z}=2z/L,\quad \mathcal{W}=w/R,\quad \ell=2R/L,$$

$$\beta=\alpha R/(L/2)^2,\quad \mathcal{F}=F(L/2)^2/(2\pi RD),$$

$$\mathcal{C}=(1-\nu^2)C(L/2)^4/(DR^2),$$

$$\mathcal{P}=Q_L(L/2)^4/DR,\quad \mathcal{P}_\mathrm{i}=Q_\mathrm{i}(L/2)^4/DR,$$

and the rest of the notation is the same as before. This was done to ensure that the equations are solved on the interval $[\![0,1]\!]$ in AUTO-07p.

When studying these equations, we fix $\mathcal{P}_\mathrm{i}=0$, which corresponds to the initial internal pressure being equal to atmospheric pressure. We take $\mathcal{I}=0.9$, $\ell=0.386$, $\nu=0.35$ to mimic our experiments, which were typically conducted using aluminium cans of radius $R=26.5$ mm and length $L=137$ mm with a volume filling fraction of approximately 90%. We determine $\mathcal{C}$ and β using Table S2, and refer to it for values of the other two nondimensional material parameters as well, i.e. γ_1 and γ_2. Finally, we solve Eq. (S19) by varying $\mathcal{P}$ and computing $\mathcal{W}$, $\mathcal{F}$ and a, which relates the level of compression to the force.

Depending on the choice of parameters, many of our solutions are strongly localised, resulting in a rapid variation of the displacement w in a narrow region of the domain and small displacements that quickly decay to zero outside this narrow region, see e.g., the black

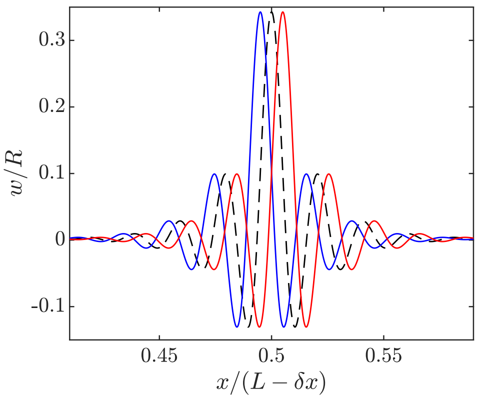

FIG. S4: **Multiplicity of strongly localised solutions.** Strongly localised solutions to Eq. (S18) computed for $\delta x/L = 0.0815$ and $F = 3300.5$ N. The (red and blue) solid curves are computed by translating the solution shown using dashed black line by ± 0.005 in $x/(L - \delta x)$ and supplying this as an initial guess to Eq. (S18).

dashed line in Fig. S4. Hence, small translation of these solutions also satisfies the equations, as demonstrated with the (blue and red) solid lines in Fig. S4. This renders parameter continuation difficult. However, the issue can be resolved by introducing additional constraints during integration, for example, by fixing the position of the undulation with the largest amplitude. We choose to impose the symmetry of solutions at $\mathcal{Z} = 0$ instead by using

$$\frac{d\mathcal{W}(\mathcal{Z}=0)}{d\mathcal{Z}} = 0, \quad \frac{d^3\mathcal{W}(\mathcal{Z}=0)}{d\mathcal{Z}^3} = 0, \tag{S20}$$

and solving Eq. (S19) on half of the total spatial domain given by $\mathcal{Z} \in [0,\ 1]$, so that only boundary conditions at $\mathcal{Z} = 1$ are enforced in addition to Eq. (S20). This limits the type of solutions that can be computed to ones with an odd number of undulations, but we have confirmed independently (by solving Eq. (S19) in MATLAB, see below) that even branches of solutions also exist.

We performed calculations with 8001 collocation points, and have tested the convergence of our results by doubling the number of points and re-obtaining results which were graphically indistinguishable from the ones presented in this paper. To begin with, we calculated all bifurcations for $\mathcal{C} = \beta = \nu = \gamma_1 = \gamma_2 = 0$ and then continued in these parameters until the values of interest were reached. We cross-validated our numerical solutions by also solving Eq. (S19) in MATLAB using second-order central differences on a numerical grid with 200 points. Moreover, this allowed us to speed up some of our calculations by pro-

viding MATLAB solutions as an initial guess to AUTO-07p before starting the parameter continuation. All numerical integrations were performed using the nondimensional version of the problem (i.e., Eq. (S19)), but the results are presented using dimensional variables for easier comparison with experiments.

Supplementary Note 7: Parameter Variation

Our theoretical model allows us to predict critical engineering strains and forces at the onset of buckling, as well as the maximum amplitudes of buckles and the strain beyond which no localised solutions can be computed. The variations of these quantities with γ_2 are discussed in the main manuscript. Here, we show how they vary with other parameters associated with the material nonlinearities i.e., γ_1 and α.

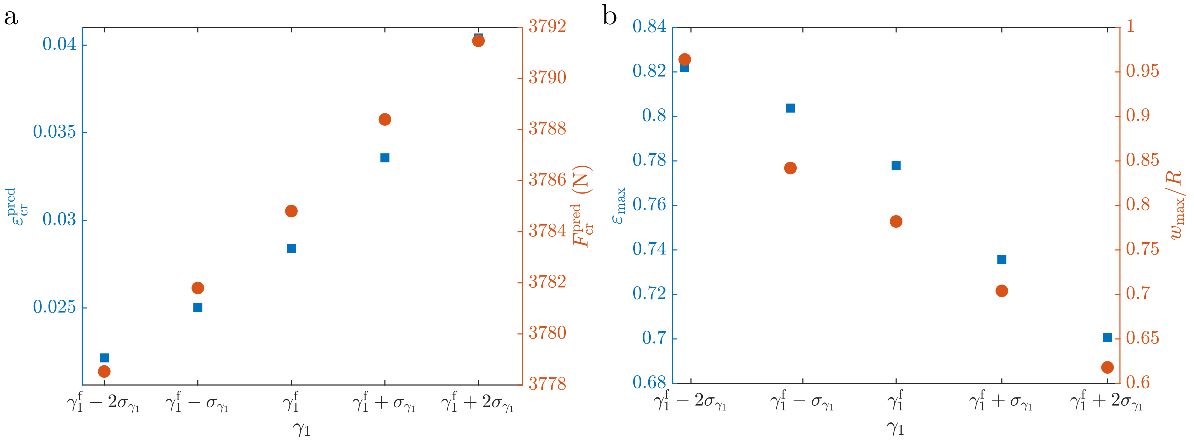

FIG. S5: **Sensitivity analysis for γ_1.** Variation in a) the predicted critical values of the engineering strain ε_{cr}^{pred} and force F_{cr}^{pred}, and b) the maximal computed strain ε_{max} and the maximal amplitude of undulations w_{max} with γ_1, where $\gamma_1^f = -2.72$ is the fitted value of γ_1 and $\sigma_{\gamma_1} = 0.17$ is the error on it, see Table S2.

In Fig. S5 we show the results of varying γ_1 in the range from $\gamma_1^f - 2\sigma_{\gamma_1}$ to $\gamma_1^f + 2\sigma_{\gamma_1}$, where γ_1^f and σ_{γ_1} are both given in Table S2 and correspond to the fitted value of γ_1 and the error on it. The trends reported for varying γ_2 (see the main manuscript) are also seen when varying γ_1. The critical engineering strain ε_{cr}^{pred} and force F_{cr}^{pred} both increase with increasing γ_1, whereas the maximum amplitude of buckles w_{max} and the maximum

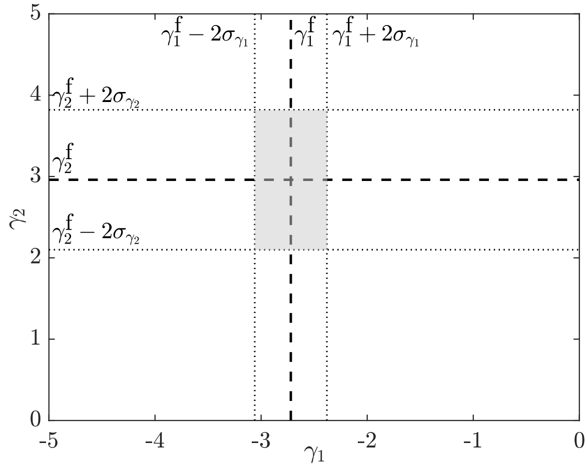

FIG. S6: **Parameter range.** The shaded region showing the range of γ_1 and γ_2 values for which the pattern formation was explored in detail. The symbols are the same as in Fig. 3 of the main manuscript and Fig. S5.

strain $\varepsilon_{\max}$ decrease. The notable difference is that γ_1^{f} is negative, which means that increasing/decreasing γ_1 actually corresponds to its magnitude decreasing/increasing. This is consistent with the interpretation of γ_1 as the "softening" nonlinearity, and γ_2 as the "re-stiffening or dissipative" nonlinearity: e.g., increasing the magnitude of γ_1 causes a decrease in the critical force, which, by contrast, increases with increasing magnitude of γ_2. Note that for all γ_1 and γ_2, which correspond to the range of parameter continuation shown in Fig. S6, the solution structure remains the same as in Fig. 3 of the main manuscript.

Different trends are seen for varying α, as shown in Fig. S7 for $\alpha^{\mathrm{f}} - 2\sigma_\alpha \leq \alpha \leq \alpha^{\mathrm{f}} + 2\sigma_\alpha$ where α^{f} is the fitted value and σ_α the corresponding error on the fit, see Table S2. Once again, α^{f} is negative, so increasing α corresponds to decreasing its magnitude. However, all quantities are now seen to decrease with decreasing magnitude of α, from the critical values of the engineering strain $\varepsilon_{\mathrm{cr}}^{\mathrm{pred}}$ and force $F_{\mathrm{cr}}^{\mathrm{pred}}$, to the maximum amplitude $w_{\max}$ and strain $\varepsilon_{\max}$. Most importantly, the observed variations are small, and none of the quantities in Fig. S7 are particularly sensitive to the small variations in α obtained in measurements of the constitutive response. Hence, we conclude that the onset of buckling as well as the post-buckling behaviour of the system are highly influenced by the material nonlinearities contributing to the hoop-stress but are less sensitive to the nonlinear contributions to the

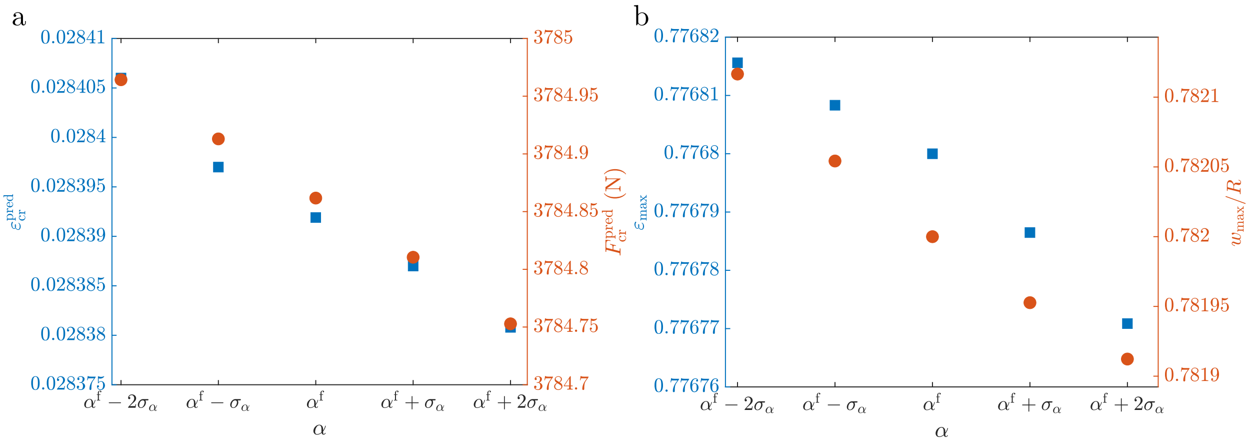

FIG. S7: **Sensitivity analysis for α.** Variation in a) the predicted critical values of the engineering strain $\varepsilon_{\mathrm{cr}}^{\mathrm{pred}}$ and force $F_{\mathrm{cr}}^{\mathrm{pred}}$, and b) the maximal computed strain $\varepsilon_{\max}$ and the maximal amplitude of undulations $w_{\max}$ with α, where $\alpha^{\mathrm{f}} = -12.84$ is the fitted value of α and $\sigma_\alpha = 0.19$ is the error on it, see Table S2.

bending.

We have also examined the effect of varying the initial pressure Q_{i}, by increasing its value from 0 to 1 atm and 2 atm. The values of the critical force $F_{\mathrm{cr}}^{\mathrm{pred}}$ decrease to 3716.1 N and 3648.5 N, respectively, whereas the corresponding critical strain values $\varepsilon_{\mathrm{cr}}^{\mathrm{pred}}$ increase to 0.033 and 0.036, respectively. Hence, the variation in Q_{i} does have a quantitative effect on data, on par with varying γ_1 within two standard deviations from the experimental mean in Table S2. However, the qualitative behaviour of the system remains unchanged, with localised solutions existing independent of the absolute value of Q_{i}.

Supplementary References

[1] D. O. Brush and B. O. Almroth, *Buckling of Bars, Plates, and Shells* (McGraw-Hill, New York, 1975).

[2] L. H. Donnell, *Stability of thin-walled tubes under torsion*, Tech. Rep. (National Advisory Committee for Aeronautics (NACA), 1935).

[3] D. Pihler-Puzović, A. Juel, G. G. Peng, J. R. Lister, and M. Heil, Displacement flows under elastic membranes. Part 1. Experiments and direct numerical simulations, J. Fluid Mech. **784**, 487–511 (2015).

[4] G. Kozyreff, P. Assemat, and S. J. Chapman, Influence of boundaries on localized patterns, Phys. Rev. Lett. **103**, 164501 (2009).
