# Supplementary material for: Sequential buckling in fluid-filled cylindrical shells
Source: Commun Phys. 2026 Mar 31;9(1):114. doi: 10.1038/s42005-026-02589-5 (PMC13098793; doi:10.1038/s42005-026-02589-5)
Supplement: Supplementary file 3 — Description of Additional Supplementary Files [file 42005_2026_2589_MOESM3_ESM.pdf]

Description of Additional Supplementary Files

File name- Supplementary Video

File description- Commercially available beverage cans deformed at different compression rates; the Supplementary Video is referenced in the manuscript.
